# Profile and satisfaction of patients with special health care needs assisted in dental specialty centers

**DOI:** 10.1590/1807-3107bor-2024.vol38.0086

**Published:** 2024-09-13

**Authors:** Saulo Vinicius da ROSA, Mariana PEROTTA, Juliana Schaia ROCHA, Renata Iani WERNECK, Sérgio Aparecido IGNÁCIO, Pablo Guilherme CALDARELLI, Paulo Sávio Angeiras de GOES, Samuel Jorge MOYSÉS

**Affiliations:** (a) Pontifícia Universidade Católica do Paraná –PUCPR, School of Medicine and Life Sciences, Curitiba, PR, Brazil.; (b) Universidade Estadual de Londrina – UEL, School of Dentistry, Londrina, PR, Brazil.; (c) Universidade Federal de Pernambuco – UFPE, Faculty of Dentistry of Pernambuco, Recife, PE, Brazil.

**Keywords:** Health Services for Persons with Disabilities, Disabled Persons, Dental Care for Disabled, Secondary Care

## Abstract

This study aimed to analyze the profile and level of satisfaction of users served in the dental specialty for patients with special healthcare needs (SHCN), based on the Brazilian Program for Improving Access and Quality (“PMAQ”) of the Centers for Dental Specialties (“CEO”). This observational, quantitative study used a national secondary database in the public domain. Data were analyzed using the chi-squared test with Bonferroni correction, Student’s t-test, and log-linear Poisson regression. Most users of the specialty “SHCN” interviewed were female (74.1% in 2014 and 68.8% in 2018), with a mean age of 41.7 (2014) and 44.9 (2018) years. For every 100 respondents who considered it regular or bad, 171 considered it good, and 199 considered it very good. Regarding satisfaction with the host of the “CEO,” there were differences between the regions of Brazil (Midwest, Northeast, Southeast, and South). There has been an increase in the number of “CEO” that serve users with autism spectrum disorder. Generally, the “CEO” network provides humanized and welcoming services, presenting better performance in the second evaluation cycle, according to user perception.

## Introduction

In Brazil, dental specialty for patients with special healthcare needs (SHCN) became official in 2002.^1^ It aims to care for people who require special dental care, either for a period of life or a lifetime. They are people with disabilities who have physical or neurological problems or both.^1-3^

People with disabilities still face barriers that hinder their dental care, recognizing beforehand that the role of the dentist who works in the health system is fundamental to overcoming these barriers.^4^ Effective healthcare implies mastering knowledge regarding the health-disease-care process, both epidemiologically and clinically, helping improve public oral health through collective interventions and personalized therapeutic projects. The fundamental assumption is to know how to interact with these people according to their health problems and the context of their lives and be aware of socio-environmental determinants, vulnerabilities, and the uniqueness of their situation.^2,5,6^

Access of the Brazilian population to the Unified Health System (SUS) should always occur through the entrance door, the health unit providing primary healthcare (PHC). This is the user’s first contact with the healthcare system, except in emergencies when the emergency room and/or hospital may be the first choice. From this first access and evaluation, the flow to the necessary comprehensive care should be coordinated by this local team, managing the referral of unresolved cases in PHC for specialized treatment through reference and counter-referral throughout the care network.^6-8^

The Ministry of Health (Ordinance No. 1,645/GM/MS, October 02, 2015) has the National Program for Improving Access and Quality in Primary Care (PMAQ-AB). It was proposed to strengthen health teams and managers to improve the quality of health services offered to the Brazilian population in municipalities through monitoring and evaluating PHC.^9^

The reorganization of the oral health field, with its progressive incorporation into existing PHC health teams, arrived at the end of 2000 with Ordinance No. 1444 from the Ministry of Health. The objective was to improve the Brazilian population’s access to oral healthcare, positively impacting epidemiological indicators and satisfaction with the clinical care received. In more complex cases, in which there is no prospect of solving an oral health problem, a referral should be made to so-called secondary (or specialized) care. The dental care unit responsible for providing resolutive care at this level of the integrated care network is the Center for Dental Specialties (“CEO”) ^10^.

The “CEO” has the mission of offering assistance in endodontics, oral diagnosis, minor oral surgery, more complex periodontics, and dentistry for patients with SHCN.^11^ Ordinance No. 599/GM/MS, of March 23, 2006, legally guaranteed specialized care for people with SHCN.^6^ The same should happen to users with systemic problems who, even with adequate drug therapy and treatment, still have difficulties in controlling chronic diseases and/or conditions such as hypertension, diabetes, metabolic syndromes, HIV, and some at-risk pregnant women.

Therefore, in addition to the PMAQ-AB, the Program for Improving the Access and Quality of Dental Specialty Centers (“PMAQ-CEO”) was created by Ordinance No. 261/GM/MS on February 21, 2013. It is an evaluation program, already conducted twice at the national level, in the cycles of 2014 and 2018, with the (voluntary) participation of the managers from “CEO”.^12,13^ The program evaluates the performance and quality of the service provided to users; those “CEO” who achieve the required quality standards are benefited with more financial resources in return of their performance.^12^

In observing the principles of transparency and public governance, with the accountability of the public investment applied to the PMAQ-CEO for society, as well as providing decision-makers and public managers with evidence of good practices, it is necessary to evaluate and compare the evaluation cycles of the program, establishing a value judgment on possible changes, whether positive or negative over the years.

This article aimed to analyze the profile and level of satisfaction of users assisted in the dental specialty for Patients with SHCN, based on the Brazilian Program for Improving Access and Quality of Dental Specialty Centers (“PMAQ/CEO”), in the first and second cycles (2014 and 2018, respectively).

## Methods

This was an observational quantitative study that used secondary databases in the public domain. These data are available on the Ministry of Health website. This data source covers the first and second cycles of the PMAQ-CEO and contains the evaluated CEO belonging to the public oral health network in Brazil in their respective geographic macroregions. The process of managing the entire database followed the appropriate and internationally recommended protocol for these cases, the RECORD Statement (https://www.equator-network.org/reporting-guidelines/record/). The PMAQ-CEO was submitted and approved by the Ethics Committee Involving Humans.^12^

For the preparation of this article, the data were filtered according to the following: a) Module I: CEO evaluation; b) Module II: Evaluation of the manager and dentist; and c) Module III: User evaluation (which could also be the parents or caregivers of patients, according to the inclusion/exclusion criteria).

The sample size varied according to the sample units of modules a, b, and c. The number of “CEO” units evaluated in the “SHCN” specialty was considered; the same establishments that participated in the first cycle (n = 874) and continued to operate in the second cycle (n = 874) were sampled. We also considered the number of valid answers and the number of “users” interviewed who were in the first (n=402) and second (n = 571) cycles.

Relevant variables were selected to know the profile and level of satisfaction of users interviewed and who had treatment in the “SHCN” specialty. They comprised the following: a) sociodemographic data: sex, age, race, housing, education, and income; b) access/use: time to reach the “CEO,” satisfaction with the specialized service provided by the team and the dentist (waiting time for service and humanized reception), and counter-reference to PHC.

Descriptive and analytical work requires complete database verification and adjustments for methodological consistency. An exploratory descriptive analysis was performed with the observation of frequency distributions, and the questions in which grades were assigned to the service in the CEO were as follows:

(1) From 0 to 10: “What grade do you give to your satisfaction with the care provided by the dentist?”

(2) From 0 to 10: “What grade do you give to your satisfaction with the service provided by professionals at the CEO’s reception?”

Questions (1) and (2) were categorized dichotomically as the cause of the low negative score (data below 8); therefore, the resulting two categories contained grades 0–8 and 9–10.

In parallel, for the questions:

(3) “How was your reception when having CEO service?” – The notes were on a 5-point Likert scale, respectively, from “very good” (1) to “very bad” (5).

(4) “In your opinion, in general, the service you receive from this CEO is?” – Idem, Likert scale. (This question refers to all the care received during the service period in the CEO).

Questions (3) and (4) were categorized as “very good” (1), “good” (2), and “regular, bad, very bad” (3) owing to the small number of answers in these options (3, 4, and 5).

From the module of interviews with users, the notes attributed by them to the care received from the dentist and the team at the “CEO” reception were selected as dependent variables. The following independent variables were entered into the final model: a) the region of Brazil where the interviewee resides, b) the level of education of the interviewees, and c) in general, the service provided by the dentist and reception team regarding reception and humanized care.

From the observation and interview modules “CEO” structure (manager and dentist), the dependent variable consisted of the types of conditions of patients treated in the “CEO.”

The chi-squared test with Bonferroni correction was used for dichotomous or polytomous nominal variables, and for continuous variables, Student’s t-test was used for independent samples, with 95% confidence and a significance level of 5%. Poisson’s log-linear regression model was used to estimate the prevalence ratios, using the “backward” method, initially incorporating all variables that presented significant differences in the univariate analysis and using the scores assigned by the user as dependent variables. The simple Poisson’s log-linear model was used to estimate prevalence when the dependent variable consisted of the types of conditions of the patients treated in the “CEO.” The point estimates of the prevalence ratios in some of the multivariate analyses are presented in the Results section based on comparisons of 100 individuals. Statistical analysis was performed using Statistical Package for the Social Sciences (IBM Statistical SPSS) version 25.0.

## Results

The majority of people assisted in the “CEO” were female (74.1% in the first and 68.8% in the second cycle) and were declared brown/mixed race in the first cycle (48.7%) and white in the second cycle (42.4%). However, there was an increase in the number of people who define themselves as having black skin color (from 8.7% to 12.7%, respectively). A balance of the sociodemographic characteristics of the interviewed sample according to univariate analysis applied to the ordinal/nominal variables is presented in Table 1.

**Table 1:** Chi-Square Test to compare the profile in the users assisted in the dental specialty for Patients with Special Needs. PMAQ-CEO 1st and 2nd cycles. Brazil, 2023.

Information about the patient served at the CEO	1^st^ Cycle (n = 402)*	%	2^nd^ Cycle (n = 571)*	%	Total (n = 973)
Sex					
Female	298a	74,1	393a	68,8	691
Male	104a	25,8	178a	31,1	282
Race/skin color (self-reported)					
White	151a	37,5	242a	42,4	393
Black	35a	8,7	73b	12,7	108
Yellow	10a	2,4	8a	1,4	18
Brown/Mestizo	196a	48,7	230b	40,2	426
Indigenous	7a	1,7	9a	1,5	16
Ignored	3a	0,7	9a	1,5	12
Do you live in this municipality (CEO headquarters where the interview was performed)?					
Yes	362a	90	522a	91,4	884
No	40a	10	49a	8,5	89
Your home is located at:					
Urban area	332a	82,5	494a	86,5	826
Rural area	69a	17,1	75a	13,1	144
Don’t know	1a	0,2	2a	0,3	3
Does the Family Health Strategy cover your home?					
Yes	306a	76,1	450a	78,8	756
No	80a	19,9	111a	19,4	191
Don’t know	16a	3,9	10b	1,7	26
How many people live in your household including you?					
1 a 3 persons	162a	40,2	280b	49	442
4 a 6 persons	213a	52,9	260b	45,5	473
7 a 10 persons	26a	6,4	26a	4,5	52
More than 10 people	1a	0,2	5a	0,8	6
What is your level of education?					
Illiterate	45a	11,1	79a	13,8	124
Literate	27a	6,7	43a	7,5	70
Incomplete elementary school	136a	33,8	180a	31,5	316
Complete elementary school	38a	9,4	43a	7,5	81
Incomplete high school	37a	9,2	54a	9,4	91
Complete high school	91a	22,6	129a	22,5	220
Incomplete undergraduate	10a	2,4	20a	3,5	30
Complete undergraduate	12a	2,9	21a	3,6	33
Postgraduate studies	6a	1,4	2a	0,3	8
What is your family income?					
No income	9a	2,2	0b	0	0
Less than 1 minimum wage	65a	16,1	83a	14,5	148
From 1 up to 2 minimum wages	205a	50,9	371b	64,9	576
From 2 up to 3 minimum wages	77a	19,1	67b	11,7	144
From 3 up to 5 minimum wages	23a	5,7	40a	7	63
From 5 up to 10 minimum wages	11a	2,7	8a	1,4	19
More than 10 minimum wages	2a	0,4	2a	0,3	4

*Chi-square test. Different letters on the same line mean statistically significant differences for each category, p < 0.05. Equal letters on the same line mean no statistical differences for each category, p > 0.05.

Table 2 shows the findings of the univariate analysis for continuous/discrete variables of people who had access and were attended by the “CEO.” Notably, the mean age increased significantly during the second cycle of the program (p < 0.05). The time required to reach the “CEO” was similar for both cycles. The same happened with the score attributed to “satisfaction” by the participants interviewed in both cycles, showing a good perception of the reception received in the “CEO” and the treatment of the dentist.

**Table 2:** Measures of central tendency and dispersion, with respective statistical significance, of variables related to people who were assisted in the dental specialty for Patients with Special Needs. PMAQ-CEO 1st and 2nd cycles. Brazil, 2023.

Access/satisfaction data	Cycle	n	Mean	SD	p-value*
Age	1^st^	400	41,67	± 15,59	0
	2^nd^	523	44,89	±15,25	
How long does it take you to reach the CEO (in minutes)?	1^st^	402	29,06	± 30,74	
	2^nd^	571	30,53	± 38,17	0,52
From zero to ten, what grade do you give for your satisfaction with the welcoming provided by the CEO’s team?	1^st^	402	9,37	± 1,29	
	2^nd^	569	9,26	± 1,70	0,28
From zero to ten, what grade do you give for your satisfaction with the care provided by the dentist?	1^st^	402	9,69	± 0,73	
	2^nd^	571	9,71	± 0,77	0,7

Parametric T Test from Student for independent samples. *Significant statistical differences when p < 0.05.

The findings of the multivariate analysis, with the score attributed by users to the “CEO,” as a dependent variable, are presented in Table 3. For every 100 users in the South region who gave scores of 9–10 to the “CEO,” there were 88 in the Southeast, 93 in the Northeast, and 78 in the Midwest (p < 0.05). Regarding education, for every 100 respondents who completed higher education or post-graduation and were classified into grades 9–10, 118 had only completed elementary school (p < 0.05). Furthermore, for every 100 users who rated the reception negatively, 160 and 176 users considered it good and very good, respectively (p < 0.05).

**Table 3:** Multivariate analysis in relation to the level of satisfaction with the service provided in the CEO (reception). PMAQ-CEO 1st and 2nd cycles. Brazil, 2023

Variable	B	SE	Wald Chi-Square	df	p-value	Prevalence ratio	95%CI
Brazilian Region							
Midwest	-0,24	0,09	7,34	1	0,01	0,78	0,66–0,93
Northeast	-0,07	0,03	4,32	1	0,04	0,93	0,88–1
North	-0,09	0,06	1,9	1	0,17	0,92	0,81–1,04
Southeast	-0,12	0,03	12,63	1	0	0,88	0,82–0,94
South	0^a^	.	.	.	.	1	.
Education							
Illiterate	0,15	0,09	2,9	1	0,09	1,16	0,98–1,38
Literate	0,14	0,09	2,63	1	0,1	1,15	0,97–1,37
Incomplete elementary school	0,17	0,08	4,08	1	0,04	1,18	1–1,39
Complete elementary school	0,1	0,09	1,17	1	0,28	1,11	0,92–1,34
Incomplete high school	0,15	0,09	2,57	1	0,11	1,16	0,97-1,38
Complete high school	0,02	0,09	0,05	1	0,82	1,02	0,86–1,21
Incomplete undergraduate	0,09	0,01	0,08	1	0,4	1,1	0,88–1,37
Complete undergraduate or Postgraduate studies	0^a^	.	.	.	.	1	.
User’s Satisfaction							
Time to start treatment at the CEO							
Up to one week	0,16	0,08	3,38	1	0,07	1,17	0,99–1,38
From one week to one month	0,18	0,08	4,4	1	0,04	1,2	1,01–1,41
Between one and three months	0,05	0,1	2,33	1	0,63	1,05	0,86–1,28
More than three months	0^a^	.	.	.	.	1	.
How was the reception at the CEO							
Very good	0,56	0,2	7,85	1	0	1,76	1,18–2,61
Good	0,47	0,2	5,31	1	0,02	1,6	1,07–2,38
Fair, bad, very bad	0^a^	.	.	.	.	1	.
In general, how do you consider the service provided at the CEO							
Very good	0,69	0,22	9,38	1	0	1,99	1,28–3,09
Good	0,53	0,22	5,64	1	0,02	1,71	1,1–2,65
Fair, bad, very bad	0^a^	.	.	.	.	1	.

Poisson Log-linear regression. ^a^Set to zero because this parameter is redundant.

The deepening of the analysis, considering the types of conditions of the patients treated in the “CEO” as dependent variables in relation to the evaluation cycles, is described in Table 4. Regarding the care of patients with autism spectrum disorder (ASD), for every 100 “CEO” managers who said they attended these patients in the second cycle, 91 said they had not done this before, with a statistical difference (p < 0.05). For patients with diabetes, patients with heart disease, and older patients, the number of managers who reported attending to these patients in the first cycle was higher than that in the second cycle (112 and 100, respectively), with p < 0.05. For every 100 CEO managers who said that they treated HIV-positive patients in the second cycle, 109 did so in the first cycle, with a statistically significant difference.

**Table 4:** Multivariate analysis in relation to PMAQ-CEO assistance for special needs, 1st and 2nd cycles. Brazil, 2023.

Variable	B	SE	Wald Chi-Square	df	p-value	Prevalence ratio	95%CI
The CEO assists patients with autism spectrum disorder (ASD)							
1^st^ Cycle	-0,91	0,01	29,38	1	0	0,91	0,88–0,94
2^nd^ Cycle	0^a^	.	.	.	.	1	.
The CEO treats patients with diabetes, heart problems and the elderly							
1^st^ Cycle	0,11	0,02	27,6	1	0	1,12	1,07–1,17
2^nd^ Cycle	0^a^	.	.	.	.	1	.
The CEO assists pregnant women and babies without any limitation							
1^st^ Cycle	0,17	0,03	23,62	1	0	1,19	1,11–1,27
2^nd^ Cycle	0^a^	.	.	.	.	1	.
The CEO assists patients with visual or hearing or speech or physical disabilities who do not have behavioral disorders							
1^st^ Cycle	0,08	0,01	22,09	1	0	1,09	1,05–1,13
2^nd^ Cycle	0^a^	.	.	.	.	1	.
The CEO assists patients with involuntary movements							
1^st^ Cycle	0	0,01	0,15	1	0,69	0,99	0,96–1,02
2^nd^ Cycle	0^a^	.	.	.	.	1	.
The CEO assists patients with HIV positive							
1^st^ Cycle	0,09	0,02	11,8	1	0	1,09	1,04–1,15
2^nd^ Cycle	0^a^	.	.	.	.	1	.
The CEO assists patients with behavior disorder							
1^st^ Cycle	-0,006	0,01	0,23	1	0,63	0,99	0,96–1,01
2^nd^ Cycle	0^a^	.	.	.	.	1	.

Simple Poisson Log-linear. ^a^Set to zero because this parameter is redundant.

## Discussion

The study analyzed the profile and level of satisfaction of users assisted in the dental specialty for patients with SHCN, based on PMAQ/CEO in its two cycles. The main findings were a high satisfaction of patients in general, with differences between some regions of the country. Among the “CEO,” there was an increase in those who attend to patients with ASD, showing the need for care for this population, and a decrease in “CEO” who serve people without behavior problems, suggesting access and resolution of care in the PHC.

By assuming the theoretical plausibility of the socio-environmental determination of the health-disease-care process, a relevant finding of the evaluation was observed as regional variation in the provision of oral healthcare for those with SHCN. There are variations among people living in the Northeast and Midwest, whose interviewees were less satisfied with the services received in the “CEO” than the residents of the South region. However, no clear pattern was observed since residents of the richer Southeast region were also more dissatisfied than residents of the South region.

Conversely, the less-educated were the most satisfied in all regions, which may reflect a problem of gratitude bias. Understandably, those who waited shorter for assistance were also more pleased. The fact that most patients did not receive (or even know what it is about) the counter-referral indicated a failure in the interaction of the points of the care network and in the longitudinality of care. This aspect drew attention as a negative highlight of this evaluation.

Some studies have evaluated patient satisfaction seen in the “CEO” in different locations in Brazil, especially in the Northeast region.^14-18^ The authors highlighted the importance of the user’s perception of the service, either in the sense of maintaining and consolidating programmatic health actions that translate into good practices or reformulating those that are not adequate, especially in access/reception until the end of the treatment. A welcoming and qualified attitude of a professional who receives patients from their arrival in the network of health services, from PHC to other levels of care, is essential and can be performed by all team members.^19-21^

The score attributed to the reception of the “CEO,” which in theory is the patient’s first contact with the specialized service, presented modest findings of some regions compared to the South region. However, in 2010, Lima, Cabral and Vasconcelos.^14^ evaluated the satisfaction of users assisted in the specialized dental services of the SUS in the capital of the Northeast region of Brazil. The questionnaire applied covered seven quality dimensions, among which was the human relationship for the work performed by the dentist. Most interviewees (64%) classified this service as excellent. The authors also emphasized that the time and attention offered by the professional during the consultation can positively influence satisfaction while facilitating humanized care.

Nora et al. categorized humanized care in PHC into three dimensions.^19^ First, the organization and infrastructure of basic health services are concerned with access, adequate treatment provision, physical environment, and clinical devices. Second, the work process involves workflow in the units, possible undesirable complications, insufficient staff, low pay, or fragmentation of work processes. Finally, the “light” technologies of human relations pertain to acceptance, bonding, qualified listening, respect, and dialogue. Humanized care is recommended for all actions and services of the SUS;^20^ therefore, it can (and should) extend to specialized services, covering the entire network. The National Humanization Policy (“HumanizaSUS”)^21,22^ reinforces the humanization and appreciation of users and knowledge of the profiles of people with disabilities or those who require specific dental care. In the specialized care of SUS, it is important to maintain and consolidate policy and innovate through links with users, managers, and employees. The findings of this study draw attention to the degree of satisfaction of patients seen in the SHCN specialty, in which the average satisfaction with the service at the reception of the “CEO” was very high in both cycles, being the same for the care offered by the dentist. Most also stated that, in general, the service was either very good or good.

In 2013, Kitamura et al.^16^ evaluated the satisfaction of patients seen in the “CEO” of the Southeast macroregion of Minas Gerais, with most patients being female aged 30–40 years. Their study found an association between satisfaction with shorter waiting time in the office and better self-perception of oral health, showing that reducing the patient’s waiting time before care is a good way to achieve a better-rated humanized service. This can be achieved through a well-routined scheduling service that has improved over the two survey cycles. In 2022, Amaral et al. investigated the association of the services provided by the “CEO” in relation to the structure and work processes with the satisfaction of the user served. There was an association between satisfaction with the work process and structure and the perception of service quality by the user.^23^

Condessa et al.^24^ described the profile of the “CEO” in their 2014 study and showed that the most reported condition is the care of patients with behavioral disorders in 98.2% of the centers. With the completion of the necessary treatment in the “CEO,” the counter-reference should be mandatory for the PHC to continue longitudinal monitoring in the service network.

The Brazilian Ministerial Guide for Oral Health Care for People with Disabilities describes the therapeutic path covered by the patient since they seek the first care in the PHC.^25^ This is the basic health unit that performs reception, listening, and anamnesis, identifying the complexity of their treatment needs in addition to health education with the participation of family members and caregivers. Only when it is not possible to solve an oral health problem should referrals to specialized care be made, which is the responsibility of the CEO.

According to the Primary Care Booklet^26,27^ of the Ministry of Health, in relation to patients with chronic diseases, pregnant women, babies, and individuals positive for HIV, in which the general state of health compromises dental treatment, these patients should have their general health stabilized in the PHC before being referred to the “CEO.” In the case of the sample under analysis, the data contrast between the first and second evaluation cycles shows a significant decrease in the number of “CEO” that assists patients who do not have problems with behavioral disorders or key limitations (people with diabetes, those with heart disease, older adults, pregnant women, and babies). This may indicate that these patients had access to and resolved their main oral health problems in PHC. Conversely, the increased number of “CEO” caring for patients with ASD, in this case, may be a desirable finding. This finding may be justified by the difficulty of assisting patients with such a profile adequately in PHC, knowing that they would be left without any care.

According to official standards in Brazil, to meet the SHCN specialty in the “CEO,” the dentist does not need to be a trained specialist, being only required the undergraduate course in dentistry. Some studies have reported the low availability of trained dentists, which also reflects the low degree of humanization in care, as a key problem.^28^ However, dentists who work in the SHCN specialty often have the knowledge and experience to serve people with disabilities, but because of the low offer of specialization courses, they do not have the opportunity or possibility of obtaining a degree. According to data from the Federal Council of Dentistry, in 2024, the official number of dentists specializing in SHCN is 925 in Brazil.^29^ Thus, even if all these professionals worked for the “CEO” of the national public network, they would not meet the need for all specialized services. This finding explains the manifest desire for continuing education or continuing education for professionals working in the SUS and for better training in SHCN care during graduation.

This study had some limitations. It is necessary to emphasize the implications of the use of secondary data, which were not collected to test previously formulated hypotheses, or to answer specific questions that arose during the research. There is a risk of classification bias or confusion along with lost data and changes in the eligibility of the respondents over time. However, the large number of sample units and individuals investigated in the two cycles allowed us to assume that many systematic errors were resolved.

## Conclusion

CEO are crucial in caring for patients with SHCN in the dental network of SUS. The main findings of this study showed a service with a high satisfaction rate, according to most interviewees, although with some structural precariousness in terms of the materials and equipment necessary for the proper exercise of the SHCN specialty in certain regions. The increase in the number of CEOs serving patients with ASD, as a prominent example, shows the relevance of specialized care to this population. The decrease in CEO assisting users without behavioral disorders but who need special care shows a path in which the resolution in primary care can be achieved satisfactorily, only triggering specialized care for users who need this type of care.

## References

[1] Fonseca ALA, Azzalis LA, Fonseca FLA, Botazzo C (2010). Análise qualitativa das percepções de cirurgiões-dentistas envolvidos nos atendimentos de pacientes com necessidades especiais de serviços públicos municipais. Rev Bras Crescimento e Desenvolvimento Hum.

[2] Dao LP, Zwetchkenbaum S, Inglehart MR (2005). General dentists and special needs patients: does dental education matter?. J Dent Educ.

[3] Marega T, Gonçalves AR, Romagnolo F (2018). Odontologia especial.

[4] da Rosa SV, Moysés SJ, Theis LC, Soares RC, Moysés ST, Werneck RI (2020). Barriers in access to dental services hindering the treatment of people with disabilities: a systematic review. Int J Dent.

[5] Mendes EV (2010). Health care networks. Cien Saude Colet.

[6] Campos MF, Souza LA P, Mendes VL (2015). A rede de cuidados do Sistema Único de Saúde à saúde das pessoas com deficiência. Interface.

[7] Starfield B (2002). Atenção Primária: equilíbrio entre necessidades de saúde, serviços e tecnologia.

[8] Cecilio LC, Andreazza R, Carapinheiro G, Araújo EC, Oliveira LA, Andrade MG (2012). Primary healthcare and the construction of thematic health networks: what role can they play?. Cien Saude Colet.

[9] Lemos LMA, Prado NMBL, Medina MG (2018). Programa Nacional de Melhoria do Acesso e da Qualidade da Atenção Básica (PMAQ-AB): modelização da política no âmbito nacional. Soc Cultura.

[10] Costa WC, Werneck MA, Palmier AC (2018). Secondary care in oral health in small municipalities: a cross-sectional evaluation of demand x access. RGO Rev Gaúch Odontol.

[11] Figueiredo N, Goes PS (2009). Development of secondary dental care: a study on specialized dental clinics in Pernambuco State, Brazil. Cad Saude Publica.

[12] Goes P, Figueiredo N, Martelli P, Luvison I, Werneck M, Ribeiro M (2018). Theoretical and methodological aspects of the external evaluation of the improvement, access and quality of centers for dental specialties program. Pesqui Bras Odontopediatria Clin Integr.

[13] Goes PS, Biazevic MG, Celeste RK, Moyses S (2022). Secondary dental care quality in Brazil: what we are talking about?. Community Dent Oral Epidemiol.

[14] Lima AC, Cabral ED, Vasconcelos MM (2010). Patient satisfaction at specialized dental clinics in Recife, Pernambuco state, Brazil. Cad Saude Publica.

[15] Lima TB (2020). Satisfação dos usuários dos centros de especialidades odontológicas do Estado da Paraíba.

[16] Kitamura ES, Bastos RR, Valente PP, Leite CG (2016). Avaliação da satisfação dos usuários dos Centros de Especialidades Odontológicas da macrorregião Sudeste de Minas Gerais, 2013. Epidemiol Serv Saude.

[17] Rosendo RA, Santos TM, Souza OE, Dantas MV, Nogueira PL, Nóbrega DR (2020). Grau de satisfação dos usuários de um Centro de Especialidades Odontológicas na Paraíba. Res Soc DeV.

[18] Costa EB, Carneiro JD, Oliveira AM (2018). Satisfação dos usuários assistidos em quatro centros regionais de especialidades odontológicas do Ceará, Brasil. Saúde Debate.

[19] Nora CR, Junges JR (2013). Humanization policy in primary health care: a systematic review. Rev Saude Publica.

[20] Santos-Filho SB (2007). Perspectives of the evaluation of Brazil's National Health Humanization Policy: conceptual and methodological aspects. Cien Saude Colet.

[21] Ministério da Saúde (BR) (2024). Política Nacional de Humanização - HumanizaSUS.

[22] Doricci GC, Guanaes-Lorenzi C (2021). [Co-management in the context of Brazil's National Humanization Policy: an integrative review]. Cien Saude Colet.

[23] Amaral JH, Vasconcelos M, Gomes VE, Werneck MA, Gaspar GD, Lopes AL (2022). User satisfaction with the secondary dental care services: is there an association between structure and work process?. Community Dent Oral Epidemiol.

[24] Condessa AM, Lucena EHG, Figueiredo N, Goes PSA, Hilgert JB (2020). Atenção odontológica especializada para pessoas com deficiência no Brasil: perfil dos centros de especialidades odontológicas, 2014. Epidemiol Serv Saude.

[25] Ministério da Saúde (BR) (2019). Guia de atenção à saúde bucal da pessoa com deficiência.

[26] Ministério da Saúde (BR), Secretaria de Atenção à Saúde, Departamento de Atenção Básica (2008). Saúde bucal.

[27] Ministério da Saúde (BR) (2018). A saúde bucal no Sistema Único de Saúde.

[28] Aguiar Viana Y, Valente JQ, Leite Vasconcelos D, Rocha EB, Lima PA, Fernandes DC (2017). Carência de profissional cirurgião-dentista especialista em pacientes com necessidades especiais. Cad Grad.

[29] Conselho Federal de Odontologia (2024). Número de especialistas em Odontologia para pacientes com Necessidades Especiais.

